# Hemophagocytic lymphohistiocytosis secondary to progressive disseminated histoplasmosis presenting as cellulitis

**DOI:** 10.1016/j.mmcr.2021.06.002

**Published:** 2021-06-24

**Authors:** Alfredo G. Puing, Shyam S. Raghavan, Maria A. Aleshin, Dora Y. Ho

**Affiliations:** a Division of Infectious Diseases, Department of Medicine, City of Hope National Medical Center, Duarte, CA, USA; b Department of Pathology, University of Virginia, Charlottesville, VA, USA; c Department of Dermatology, Stanford University School of Medicine, Stanford, CA, USA; d Division of Infectious Diseases and Geographic Medicine, Department of Medicine, Stanford University School of Medicine, Stanford, CA, USA

**Keywords:** *Histoplasma*, Histoplasmosis, Cellulitis, Hemophagocytic lymphohistiocytosis, Lupus

## Abstract

Histoplasmosis-associated hemophagocytic lymphohistiocytosis is a rate but lethal disease in immunocompromised hosts. Unusual clinical presentations make diagnosing invasive fungal infection even more challenging. Here we present a case of hemophagocytic lymphohistiocytosis secondary to progressive disseminated histoplasmosis presenting as cellulitis in a patient with systemic lupus erythematous. A high index of suspicion combined with histopathology and molecular diagnostic techniques are important to establish an accurate and timely diagnosis of opportunistic infections in immunocompromised patients.

## 1 Introduction

Hemophagocytic lymphohistiocytosis (HLH) secondary to disseminated histoplasmosis is a rare but potentially lethal disease of highly immunocompromised patients that presents with high fevers, elevated ferritin, hepatosplenomegaly, cytopenias and coagulopathy [1]. Mostly seen in patients with Human Immunodeficiency Virus/Acquired Immunodeficiency Syndrome (HIV/AIDS), histoplasmosis-associated HLH has been less frequently described in patients with systemic lupus erythematous (SLE) [2]. Disseminated histoplasmosis can present with a variety of cutaneous lesions, with cellulitis being a less common but recognized dermatologic manifestation [3].

## 2 Case presentation

A 25-year-old woman originally from Mexico with SLE diagnosed 5 years prior and subsequently complicated by biopsy-proven lupus nephritis class III, presented to the emergency department with epistaxis and subjective fever for one week, fatigue and lower abdominal pain. Immunosuppressive regimen upon presentation included mycophenolate, hydroxychloroquine and prednisone. Her prednisone dose had been increased from 20 to 60 mg/day five months earlier due to high grade proteinuria, then decreased to 40 mg/day for two weeks prior to presentation.

**Fig. 1:**
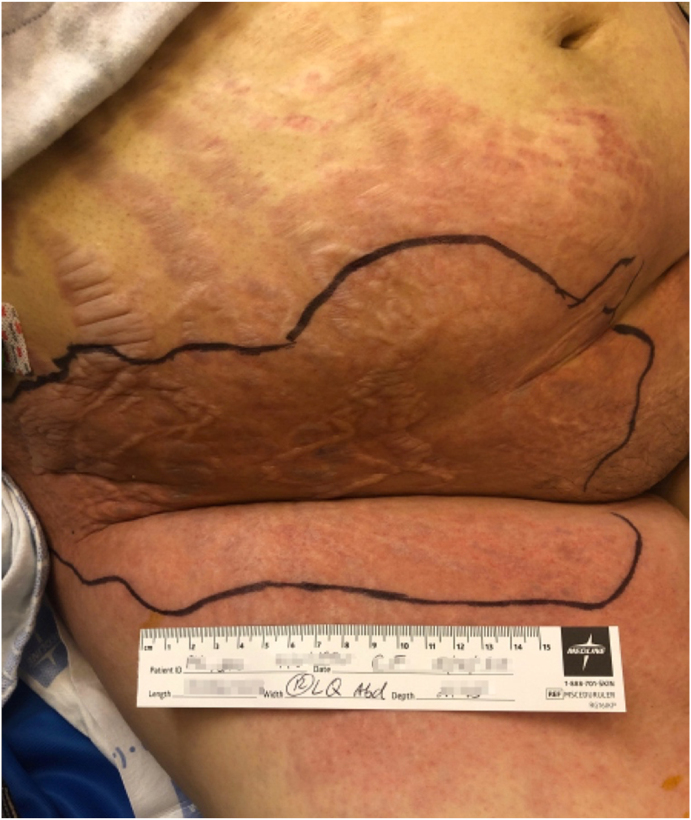
Prominent edematous striae and extensive pink indurated plaque on the right lower abdomen. (For interpretation of the references to colour in this figure legend, the reader is referred to the Web version of this article.)

On initial evaluation (day 0), the patient had a fever of 100.3 °F but was otherwise hemodynamically stable. Physical examination revealed moon facies, mild malar erythema, conversational dyspnea with lungs clear to auscultation, and lower abdominal tenderness. Skin examination demonstrated a tender pink indurated plaque extending from the right lower quadrant of the abdomen to the right anterior thigh, with overlying prominent edematous striae (Fig. 1). There were also diffuse striae on her trunk and lower extremities, as well as an ecchymotic plaque on her right medial shin and an adjacent ecchymotic subcutaneous soft nodule.

Laboratory studies on admission demonstrated pancytopenia with a leukocyte count of 3.2 × 10^9^/L, hemoglobin level of 8.3 g/dL and platelet count of 27 × 10^9^/L, kidney injury with a creatinine of 2.28 mg/dL, elevated alanine aminotransferase level of 85 U/L and aspartate aminotransferase level of 271 U/L with normal bilirubin levels, prolonged coagulation times with prothrombin time of 15.1 seconds and partial thromboplastin time of 35.6 seconds, INR of 1.2, fibrinogen of 400 mg/dL, and remarkably elevated ferritin to 78,072 ng/mL. A CT scan of her chest, abdomen and pelvis on day 1 showed nodular peribronchial vascular opacities throughout the lungs suspicious for an atypical infectious process, and a diffuse anterolateral abdominal wall stranding without intraabdominal abnormality.

**Fig. 2:**
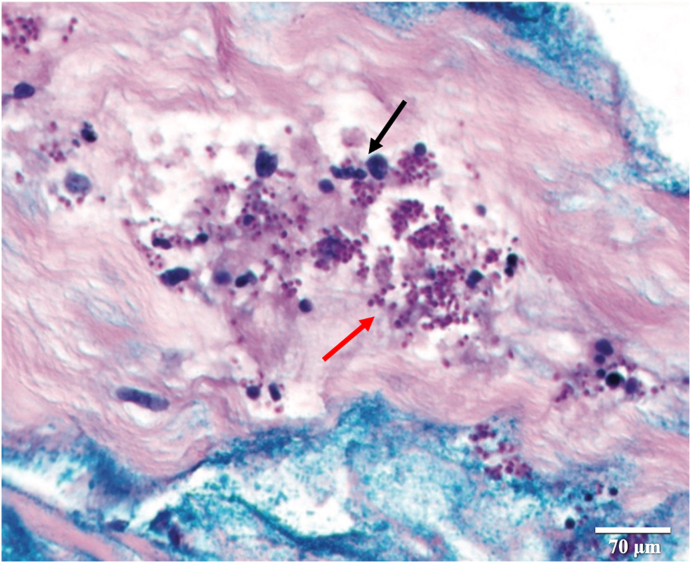
High power periodic acid-Schiff stain of the abdominal skin biopsy revealed a perivascular infiltrate of histiocytes (black arrow) with scattered small yeast forms (red arrow). (For interpretation of the references to colour in this figure legend, the reader is referred to the Web version of this article.)

A skin biopsy done on the first day of admission revealed a perivascular infiltrate of histiocytes (Fig. 2, black arrow) with scattered small yeast forms (Fig. 2, red arrow). Antifungal treatment was initiated on day 2, consisting of intravenous fluconazole 400 mg loading dose followed by 200 mg daily. A peripheral blood smear to evaluate pancytopenia demonstrated yeast forms within monocytes suggestive of disseminated histoplasmosis. In light of this, fluconazole was switched to intravenous liposomal amphotericin B (L-AmB) 5 mg/kg on day 4. Fungal culture from the skin biopsy, grown on Saboraud's dextrose agar, showed more than 100 yeast colonies closely resembling *Histoplasma capsulatum* which was confirmed by real-time polymerase chain reaction amplification and sequencing of the internal transcribed spacer 2 and D2 region of the fungal 28s ribosomal RNA gene. A diagnosis of HLH secondary to disseminated histoplasmosis was made on day 4 after the patient met five out of the eight HLH-2004 criteria (fever ≥38.5 °C, pancytopenia, fasting triglycerides 509 mg/dL, ferritin 78,072 ng/mL, soluble IL-2 receptor alpha 28,440 pg/mL [4]). On day 5, patient was started on HLH-specific therapy based on HLH-94 protocol with renally-dosed etoposide twice weekly for the first two weeks and once weekly thereafter through week eight, and intravenous dexamethasone taper started at 10 mg/m^2^ daily. Over the course of her hospitalization, her clinical status continued to decline, and she developed multiorgan failure requiring continuous renal replacement therapy and mechanical ventilatory support. MRI of the head on day 9 revealed multifocal supratentorial and infratentorial subcortical and cortical acute infarcts. Despite antifungal and HLH-specific therapies, the patient was transitioned to comfort care and died from multi-organ failure 45 days after admission.

## 3 Discussion

Histoplasmosis is an endemic mycosis caused by the dimorphic fungus *Histoplasma capsulatum*. Although histoplasmosis is found worldwide, *Histoplasma capsulatum* var. *capsulatum* is endemic in tropical areas of Central America and South America. In the United States, infection is most common in the Ohio and Mississippi river valleys [5,6]. In contrast, *Histoplasma capsulatum* var. *duboisii* is an invasive fungal infection endemic in central and west Africa [7].

Infection develops by inhalation of *Histoplasma* microconidia into the lungs. Once in the alveoli, conidia are engulfed, but not killed, by neutrophils and macrophages where they partially, if not entirely, covert into the yeast form [8]. From there, yeasts migrate, presumably intracellularly within macrophages, to the adjacent draining lymph nodes and subsequently throughout the reticuloendothelial system (e.g., liver, spleen, lymph nodes, and bone marrow) by hematogenous spread. Cell-mediated immunity to *H. capsulatum* develops approximately 10–14 days after exposure and is critical to bring the infection under control. In healthy subjects, dissemination is usually clinically silent and most infections are asymptomatic. However, failure to activate the macrophage fungicidal capacity appears to be crucial in patients who develop pulmonary and progressive disseminated histoplasmosis (PDH). Individuals at higher risk for developing PDH include people living with HIV/AIDS or other immunodeficiency, chronic steroid users, solid organ transplant recipients in antirejection therapies, and patients receiving tumor necrosis factor-alpha inhibitors [9]. In our case, we suspect that high-dose prednisone along with SLE were the main risk factors for dissemination. PDH can also occur from reactivation of latent infection (due to persistence of viable organisms in the tissues following primary infection), after reinfection (following a massive exposure to *H. capsulatum*), or in patients at the extremes of age (infants and older people) [10]. Cutaneous lesions have been described in 10–15% of cases of PDH, and can manifest as papules, plaques, ulcers, and, rarely, cellulitis. Skin lesions may also occur as primary cutaneous histoplasmosis by direct inoculation of spores through direct trauma to the skin, particularly thorn prick, which was not suspected in this case since the patient did not recall a specific event. In our patient, cellulitis was rather a manifestation of PDH after likely an inhalation exposure to *H. capsulatum* which has been rarely described in patients with SLE [3,11].

HLH is a life-threatening severe systemic inflammatory syndrome with characteristic signs of fever, hepatosplenomegaly, cytopenias and coagulopathy [1]. Primary (familial) HLH is most frequently seen in children with mutations affecting the function of natural killer (NK) cells and cytotoxic T lymphocytes (CTLs) which leads to poor clearance of infected cells, uncontrolled macrophage activation, and excessive production of interferon gamma and other cytokines with resulting end-organ damage. Secondary (acquired) HLH occurs in adults and is commonly triggered by infections, malignancies, or rheumatologic disorders [2]. The pathogens currently described as triggers of HLH are either intracellular or facultatively intracellular, which include mainly viruses (Epstein-Barr virus, cytomegalovirus, HIV), but also bacteria (*Babesia*, *Coxiella, Listeria*), mycobacteria, parasites (*Leishmania*, malaria) and fungi. *Histoplasma* is a recognized but rare cause of HLH [12]. In a recent review of the literature, Jabr et al. reported 65 cases of histoplasmosis-associated HLH where the most common underlying immunosuppressive condition was HIV/AIDS [13]. Three previous case reports have described HLH secondary to disseminated histoplasmosis in SLE [[14], [15], [16]]. Intracellular microbial pathogens are particularly prone to trigger HLH, because NK cells are essential for clearance of infected cells and activated CTLs. Without proper functioning of NK cells, a patient suffers from a double defect of an exuberant but ineffective immune response that damages the host without clearing the infection [13].

The diagnosis of HLH can be established if there is a molecular diagnosis consistent with HLH, or if a patient meets at least five of the following eight diagnostic criteria: fever, splenomegaly, cytopenia of at least 2 cell lines, hypertriglyceridemia and/or hypofibrinogenemia, hemophagocytosis on pathology, low or no NK cell activity, ferritin ≥500 ng/mL, or increased soluble IL-2 receptor (e.g., sCD25) ≥2400 U/mL [4]. Although disseminated histoplasmosis is a recognized cause of high fevers, elevated ferritin, hepatosplenomegaly, and pancytopenia, this case emphasizes the need to also consider HLH in this clinical scenario.

Treatment of the triggering condition is recommended as first line therapy for patients with secondary HLH [17]. Antifungal treatment for PDH is based on disease severity and the presence or absence of central nervous system (CNS) involvement. For patients with mild to moderate symptoms, guidelines recommend the use of itraconazole (200 mg 3 times daily for 3 days and then twice daily for at least 12 months). Whereas in patients with moderately severe to severe disease, including patients with CNS involvement, L-AmB (3–5 mg/kg daily) is recommended followed by oral itraconazole once the patient has been stabilized [18,19]. Indications for initiating HLH-specific chemotherapy is less clear in the adult population with the acquired (rather than genetic) form of the disease. Chemotherapy regimens recommended for HLH are frequently based on pediatric protocols, such as HLH-94 and HLH-2004, which consist of corticosteroids, typically dexamethasone, cyclosporine A, intrathecal therapy, and etoposide, to delete activated T cells and suppress inflammatory cytokine production [4,17]. In our patient, treatment with intravenous L-AmB was initiated after the skin biopsy identified yeast forms suggestive of *Histoplasma,* and HLH-specific therapy with etoposide and dexamethasone was started once the patient met five out of the eight HLH-2004 criteria, in an attempt to control the infection while abating an exaggerated immune response. Unfortunately, the patient succumbed to the disease 45 days after hospital admission. The 30- and 90-day mortality rates for histoplasmosis-associated HLH have been reported in a recent case series to be 46% and 64%, respectively [20].

Our case highlights the importance of skin biopsy, histopathology and molecular diagnostic studies for an accurate and timely diagnosis of opportunistic infections in immunocompromised hosts who may have uncommon clinical presentations, and the need to have a high index of suspicion for HLH in patients presenting with disseminated histoplasmosis and vice versa, as these two conditions share clinical and laboratory features, but demand different targeted treatments.

## Declaration of competing interest

There are none.
